# Haplogroup Structure and Genetic Variation Analyses of Mitochondrial Genome SNPs in the Iranian Population

**DOI:** 10.34172/aim.33639

**Published:** 2025-03-01

**Authors:** Masoumeh Ghasemi, Marzieh Mohseni, Zohreh Fattahi, Masoud Edizadeh, Maryam Beheshtian, Fatemeh Keshavarzi, Khadijeh Jalalvand, Mohammadamin Omrani, Ali Khanbazi, Yasser Riazalhosseini, Mohammad Reza Akbari, Kimia Kahrizi, Hossein Najmabadi

**Affiliations:** ^1^Genetics Research Center, University of Social Welfare and Rehabilitation Sciences, Tehran, Iran; ^2^GENOKS Genetic Disease Diagnostic Center, Ankara 06560, Turkey; ^3^Urology and Nephrology Research Center (UNRC), Shahid Beheshti University of Medical Sciences, Tehran, Iran; ^4^Kariminejad - Najmabadi Pathology & Genetics Center, Tehran, Iran; ^5^Victor Phillip Dahdaleh Institute of Genomic Medicine at McGill University, Montreal, Quebec, Canada; ^6^Institute of Medical Science, Faculty of Medicine, University of Toronto, Toronto, Canada

**Keywords:** Genomic diversity, Haplogroup, Mitochondrial DNA, Targeted mtDNA sequencing method, Whole exome sequencing

## Abstract

**Background:**

Mitochondrial DNA (mtDNA) is a valuable marker for population studies and forensic investigations. Recent advancements in massively parallel sequencing technologies enable whole mitochondrial genome sequencing. This study collected blood samples from unrelated Iranian participants from four ethnic groups: Persian, Kurd, Lur, and Azeri. We mapped mtDNA haplogroups according to genetic ancestry and investigated the ethnic similarities within the Iranian population.

**Methods:**

Complete mtDNA sequences were generated with targeted mtDNA sequencing method and haplogroups were determined on the base of mitogenome polymorphisms. Additionally, we used data from the whole exome sequencing (WES) of the current samples to compare the variants identified by two different mitochondrial testing methods. Principal component analysis (PCA) calculations were performed using the R software to determine diversity between unrelated individuals of various ethnicities.

**Results:**

A total of 129 sub-haplogroups were identified in 15 main haplogroups. The findings revealed high frequencies of haplogroups U and H (22.4% and 20.3%, respectively) in the Iranian population. The PCA scatter plots revealed overlapping diversity, with no distinct trends separating the groups in these four groups within the Iranian population. In the present samples, the WES method identified only 57.8% of the variants detected by the targeted mtDNA sequencing method.

**Conclusion:**

Variant studies do not show much difference, which indicate a small genetic difference between the central ethnic groups of Iran. Furthermore, comparing the targeted whole mitochondrial genome to mitochondrial data from WES in our study samples highlights the notion that targeted entire mitochondrial genome is a gold standard method for variant detection.

## Introduction

 Human mitochondrial DNA (mtDNA) is a circular duplex and consists of 16 569 base pairs (bps).^1^ mtDNA variants are maternally transmitted without undergoing recombination, allowing for their accumulation over successive generations. This characteristic of mtDNA has made it a popular tool for studying population genetics, phylogenetic evolution, human migration, and medical and forensic studies. Many studies on mtDNA analysis have been published.^2-11^ A mitochondrial haplogroup includes individuals with identical accumulated mtDNA variants, typically found in specific geographic regions and can be traced through maternal lineage. These haplogroups constitute distinct branches within the mitochondrial phylogenetic tree. Certain haplogroups are predominantly associated with specific geographic regions. Haplogroups L0–L6 are commonly found among Sub-Saharan Africans, while R5–R8, M2–M6, and M4–67 are prevalent in South Asian populations. Similarly, haplogroups A–G, Z, and M7–M9 are typically observed in Europeans, East and Southwest Asians. North African populations predominantly share haplogroups HV, U, T, J, X, N1, and N2.^12-14^

 Some laboratories perform Sanger sequencing on a small portion of the mtDNA, specifically the non-coding hypervariable regions (HVI-HVIII).^15,16^ Attempting to improve the effectiveness of mtDNA in human identification, recent studies over the past decade have expanded their analyses to include the entire mtDNA genome using next generation sequencing (NGS).^17-19^ Also, studies have shown that off-target exome sequencing and whole-genome sequencing (WGS) effectively target both mtDNA and nuclear DNA. These approaches are useful for diagnosing monogenic cases and conducting association studies for multifactorial disorders.^20-24^ Similar studies include the following: Wagner et al, and Delmiro et al assessed the feasibility of performing mtDNA analysis using exome data. They used mtDNA sequences extracted from exome data to reconstruct human population history, employing mtDNA variants as markers, and to explain the role of mtDNA in pathology.^20,25^ Patowary et al examined mtDNA sequences obtained from whole-exome sequencing to investigate the association of variants and haplogroups with autism spectrum disorder. Nevertheless, to our knowledge, the literature lacks evidence comparing the efficiency of mitochondrial variant calling from whole-exome data with that from whole mitochondrial sequencing data.^26^

 Iran’s location in southwest Asia makes it an ideal place for studying human diversity, history, and origins. It also plays a crucial role in migrations between populations in West Asia and beyond. In recent decades, molecular genetic methods, including the analysis of matrilineal mtDNA variability, have been widely used to reconstruct histories of various ethnic groups and other populations. These studies have aimed to shed light on the genetic diversity, population movements, and expansion patterns that have influenced the Iranian population. It is important to mention that most of these mtDNA researches have utilized low-resolution techniques to uncover genetic variation, such as examining mtDNA haplogroup-specific sites and hypervariable regions. Whole mitogenome sequencing substantially enhances discrimination power, making it a highly informative source for studying female-specific aspects of demographic history.^2^ To gain a deeper insight into mtDNA variability among Iranians, we present extensive mtDNA diversity data from four populations in the western and central regions of Iran. These populations represent the most prevalent segments of the Iranian population, analyzed through mitogenome sequencing. Additionally, we compared our findings to mitochondrial variants identified by analyzing off-target whole exome data from the Iranian population to compare the power of these two methods for mitochondrial variant detection.

## Materials and Methods

###  Sampling and DNA Extraction

 Blood samples were collected from unrelated individuals from different ethnic groups in Iran. These samples are stored in the Iranome Center at the University of Welfare and Rehabilitation Science. The DNA samples were taken from the Iranome Project, as mentioned in a previous study.^27^ The sample size was determined using Morgan’s formula (n = z^2^ pq/d^2^) with parameters d = 0.05, p = q = 0.5, and z = 1.96, resulting in at least 42 individuals per ethnic group. Approximately 50 samples were selected from each ethnic group. After quality control steps, the final sample count for statistical analysis included 52 Persians, 46 Kurds, 50 Lurs, and 44 Azeris. DNA samples of one individual per family were included; most of the cases were above 40 years of age. They were not affected by known genetic disorders and did not have a history of such disorders in their two previous generations. Genomic DNA was isolated through a conventional salting-out method.

###  Analysis of mtDNA Sequences with Targeted Whole mtDNA Sequencing and Whole-Exome Sequencing Data

 Isolated genomic DNA was used for the whole mtDNA assay. DNA libraries were prepared following the CleanPlex Mitochondrial disease kit from Paragon company, which involved two steps: multiplex PCR and indexing PCR. We generated complete mitochondrial genome sequence data using an Illumina MiSeq sequencer (Illumina, San Diego, USA). Whole mtDNA sequences were analyzed using Illumina pipelines and compared to the revised Cambridge Reference Sequence (rCRS).^28^ The mtDNA sequences obtained from these individuals were utilized as quality control samples to identify mtDNA variants from exomic sequences. Raw paired-end reads (100 bp) from targeted mtDNA sequencing were mapped to the human genome assembly GRCh38 using the Burrows-Wheeler Aligner (BWA-MEM version v07-17) with default mapping options.^29^ The mtDNA genome according to the rCRS consists of 16,569 base positions. Duplicate reads were eliminated using Picard version 2.20.21. The rCRS for human mtDNA, as reported by Andrews et al and accessible in the GenBank NCBI database under accession number NC_012920.1, was extracted using SAMtools version 0.1.19. The average mtDNA coverage was computed using the Genome Analysis Toolkit (GATK) version 3.8-1-0.^28,30,31^ For each sample, mtDNA BAM files were created. Subsequently, we applied the DRAGEN germline Pipeline to extract mitochondrial BAM files to produce variants in the variant call format (VCF) for each sample. Variant annotation was performed using the Ilyome software. In the subsequent analysis, we utilized data from previous whole exome sequencing (WES) studies to compare targeted mtDNA variants with those identified in WES. The same samples from WES data were genotypically characterized using the Axiom Precision Medicine Research Array (PMRA) by Life Technologies in a previous project of this center to analyze mitochondrial variant calling. The target exome files encompass the same mtDNA regions, with each target segment averaging 1000 base pairs. MtDNA sequences were isolated from these individuals, and variants were called using the Sentieon DNAseq pipeline.^32^ Only uniquely mapped reads with quality scores of at least twenty were retained to identify mtDNA variants.

###  Haplogroup Classification

 We used raw VCF files for the entire mtDNA from 192 samples of Iranian individuals to identify their maternal haplogroups. Haplogroup classification was performed using HaploGrep 2.0,^33^ referencing PhyloTree Build 17.^34^

###  Statistical Analysis 

 Haplogroup frequency was estimated per ethnic group. To analyze the similarity among the subpopulations between any of the four groups, principal component analysis (PCA) with a Varimax rotation method was independently performed using R (version 3.6.1, cran-project.org). This method helps simplify the interpretation of the principal components by maximizing the variance of the squared loadings of a factor across variables, making it easier to identify patterns and differences among ethnic groups.

## Results

 In our study, the mean depth of coverage for mitochondrial sequences was ~1000x and data from previous whole-exome off-target reads was ~100x. A total of 910 mtDNA single-nucleotide variants (SNVs) and insertion/deletion (INDEL) polymorphisms were identified among the 192 samples with either method. Comparison between mitochondrial variants identified in whole-exome data and those found in targeted mtDNA sequencing for the same samples showed that 526 variants, or 57.8%, were shared, and 384 certain mtDNA variants (including MT: 7861-T-C, and MT:72-T-A) were detected only in targeted whole mtDNA data. Additionally, 10 novel variants were identified in the current study that have not been documented in any mitochondrial population databases, such as Mitomap, Hmtdb, and WGS. All of these variants were detected with the targeted mitochondrial sequencing method and are shown in Table 1. Six of the newly discovered variants can be pathogenic and disease-related variants based on *in silico* analyses. However, none of the variants have been clinically confirmed as pathogenic mutations.

**Table 1:** New Variants Identified by Targeted Sequencing of the Whole Mitochondrial Genome

**Input**	**HGVS_g**	**AF_ WGS**	**AF_ mitomap**	**AF_ gnomad**	**AF_ Helix**	**HmtDB_ Pathogenicity**	**ACMG_ Classification**	**HmtDB_ DiseaseScore**	**Homoplasmy**	**Heteroplasmy**	**Lucos**
MT:g.3186T > A	NC_012920.1:m.3186T > A	—	—	—	—	—	VUS	—	—	0.1%	MT-RNR2
MT:g.5624C > A	NC_012920.1:m.5624C > A	—	—	—	—	Pending Classification	VUS	0	—	31%	MT-TA
MT:g.6849A > C	NC_012920.1:m.6849A > C	—	—	—	—	Pathogenic	VUS	0.831	—	34%	MT-CO1
MT:g.7411A > C	NC_012920.1:m.7411A > C	—	—	—	—	Pathogenic	VUS	0.841	—	27%	MT-CO1
MT:g.8129A > C	NC_012920.1:m.8129A > C	—	—	—	—	Pathogenic	VUS	0.611	—	28%	MT-CO2
MT:g.8132A > C	NC_012920.1:m.8132A > C	—	—	—	—	Pathogenic	VUS	0.508	—	26%	MT-CO2
MT:g.10571A > G	NC_012920.1:m.10571A > G	—	—	—	—	—	VUS	—	100%	—	MT-ND4L
MT:g.13706T > C	NC_012920.1:m.13706T > C	—	—	—	—	Pathogenic	VUS	0.886	—	37%	MT-ND5
MT:g.15270T > C	NC_012920.1:m.15270T > C	—	—	—	—	Pathogenic	VUS	0.89	—	0.4%	MT-CYB
MT:g.15940DelT	NC_012920.1:m.15940DelT	—	—	—	—	—	—	—	—	96%	MT-TT

###  mtDNA Haplogroup Frequency Profile

**Figure 1:**
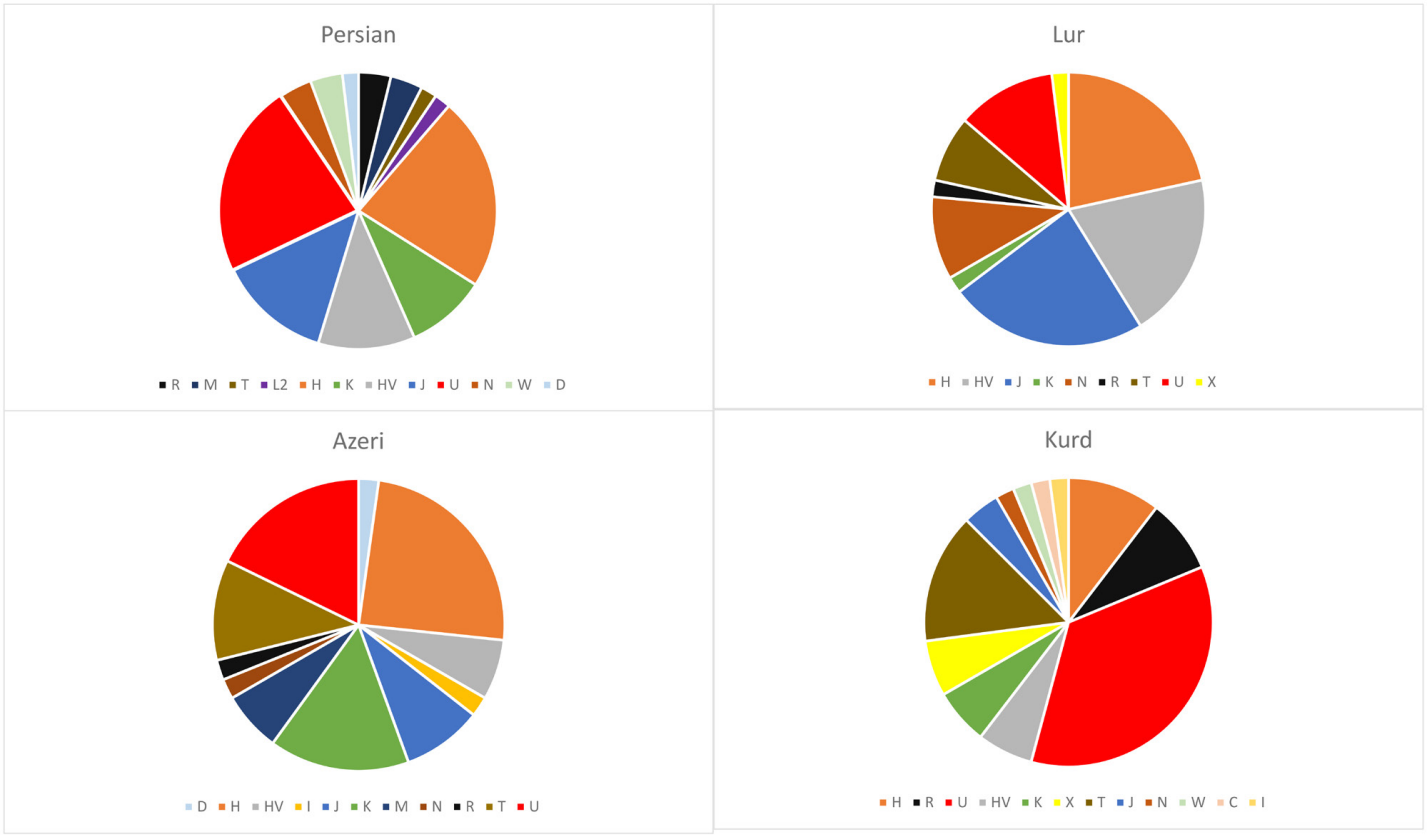
Mitochondrial haplogroup frequencies of 192 individuals from four ethnic groups in Iran

 The haplogroup assignments for Azeris, Persians, Kurds, and Lurs, and the entire Iranian mtDNA data set according to the classification system of Phylotree build 17^34^ are presented in Table 2. A total of 129 distinct sub-haplogroups or paragroups (unspecified sublineages within a clade) were identified, all of which belong to 15 main haplogroups. Maternal haplogroups (H, U, HV, K, J, M, N, L, R, T, W, I, D, C, and X) were detected from mitochondrial variants obtained through targeted whole-mtDNA sequencing. The predominant haplogroups in the Iranian population are U (22.4%) and H (20.3%) followed by J1, HV, and T (Figure 1). Every ethnic group was found to have prominent Eurasia and European haplogroups, with Azeris and Persian ethnic groupings having prominent Asian haplogroups as well. Additionally, African/African American in the Persians group, and Latino/admixed American population in Kurds were found (2.2%). Table 2 shows the frequencies of each haplogroup in the ethnic groups. The findings suggested that the frequencies of haplogroups H and U differed between Kurd and other groups, whereas haplotype H was more frequent in all groups, it had minor frequency in the Kurd group. Moreover, haplotype U was more frequent in the Kurd group than in other groups.

**Table 2:** Mitochondrial Haplogroup Frequencies in Four Ethnic Groups of the Iranian Population

**Haplogroup/ Ethnics**	**Azeri**	**Lur**	**Persian**	**Kurd**	**Total**
H*	4	4	0	0	8
H1ak	1	1	1	0	3
H2A	1	0	1	0	2
H2a1	0	1	0	0	1
H4a	1	0	0	0	1
H5	0	1	0	0	1
H13a	0	1	1	0	2
H14b3	0	1	0	0	1
H15a1a	1	0	0	0	1
H15a1b*	0	1	0	0	1
H47	2	1	0	0	3
H107	1	0	0	0	1
H92	0	0	3	0	3
H1c22	0	0	1	0	1
H1 + 16355	0	0	1	0	1
H13a2a1	0	0	1	0	1
H2a1a	0	0	1	0	1
H101	0	0	1	0	1
H + 152	0	0	1	0	1
H20	0	0	0	3	3
H5 + 16192	0	0	0	1	1
H13a1a2	0	0	0	1	1
H (Total)	11(5.7%)	11(5.7%)	12(6.3%)	5(2.6%)	39(20.3%)
HV	2	1	2	0	5
HVa1	1	1	0	0	2
HV1a1	0	2	1	0	3
HV2a	0	1	0	0	1
HV14	0	2	0	1	3
HV18	0	2	0	0	2
HV19	0	0	1	0	1
HV1a3a	0	0	0	1	1
HV2a2	0	0	1	0	1
HV2a1	0	0	1	0	1
HV13b	0	0	1	0	1
HV (Total)	3(1.5%)	9(4.7%)	7(3.6%)	2(1.0%)	21(11.0%)
J1	0	0	0	1	1
J1b1b	2	4	0	0	6
J1b	0	2	0	0	2
J1b1a2a	1	0	0	0	1
J1b1b1a	0	1	0	0	1
J1b1a1 + 146	0	1	0	1	2
J1c	0	1	0	0	1
J1c2	1	0	0	0	1
J1d3a2	0	1	0	0	1
J1 + 16193	0	2	3	0	5
J1b2	0	0	1	0	1
J1b1a1	0	0	1	0	1
J1c8	0	0	1	0	1
J1b3b1	0	0	1	0	1
J (Total)	4(2.0%)	12(6.3%)	7(3.6%)	2(1.0%)	25(13.0%)
K1	0	1	1	0	2
K1a7	1	0	0	0	1
K1a4f	1	0	0	1	2
K1a + 150	1	0	0	0	1
K1a17	1	0	0	0	1
K1a23	1	0	0	0	1
K1b1c	1	0	0	0	1
K1a	0	0	1	1	2
K1a3	0	0	1	0	1
K1a + 195	0	0	0	1	1
K (Total)	6(3.2%)	1(0.5%)	3(1.6%)	3(1.6%)	13(7.0%)
U1a1a	0	1	1	0	2
U2e1a1	1	0	0	0	1
U3b	1	0	0	0	1
U5a2d	1	0	0	0	1
U7	1	0	0	1	2
U7a	1	3	1	3	8
U7a1 + @16192	1	0	0	0	1
U7a3	0	1	0	0	1
U7a4	1	0	1	0	2
U7a4a	1	0	0	0	1
U8b1a1	0	1	0	0	1
U7b	0	0	1	1	2
U1a1a3	0	0	1	0	1
U1a1c1c	0	0	1	0	1
U8b1a2b	0	0	1	1	2
U1a1c1d	0	0	1	0	1
U7a4a1	0	0	1	0	1
U2e	0	0	1	0	1
U2e1'2'3	0	0	1	0	1
U4a	0	0	1	1	2
U4a1	0	0	0	1	1
U7a2a	0	0	0	1	1
U7a3a*	0	0	0	1	1
U7a4a1a	0	0	0	2	2
U5a1	0	0	0	1	1
U8b1b2	0	0	0	1	1
U7a1a1	0	0	0	1	1
U8b1a2a	0	0	0	1	1
U1a1c1	0	0	0	1	1
U (Total)	8(4.1%)	6(3.1%)	12(6.2%)	17(8.8%)	43(22.4%)
T1a	1	0	0	1	2
T1a2	1	0	0	0	1
T1a8a	0	1	0	0	1
T2	1	0	0	1	2
T2a	0	1	0	0	1
T2a3	0	1	0	0	1
T2b34	0	1	0	0	1
T2g	1	0	0	0	1
T2j1	1	0	0	0	1
T2b3 + 151	0	0	1	0	1
T1b3	0	0	0	1	1
T1b4	0	0	0	1	1
T2d1	0	0	0	1	1
T2b	0	0	0	1	1
T2b4h	0	0	0	1	1
T1a1b1	0	0	0	1	1
T (Total)	5(2.6%)	4(2.1%)	1(0.5%)	8(4.2%)	18(9.4%)
M3a1 + 204	0	0	1	0	1
M6a1a	1	0	0	0	1
M7b1a1a1	1	0	0	0	1
M8a2c	1	0	0	0	1
M18c	0	0	1	0	1
M (Total)	3(1.5%)	0(0.0%)	2(1.0%)	0(0.0%)	5(2.6%)
N	0	0	1	0	1
N1a1'2	0	1	0	0	1
N1b1a2	0	0	1	0	1
N1b1a3	1	0	0	0	1
N2a2	0	0	0	1	1
N3a	0	4	0	0	4
N (Total)	1(0.5%)	5(2.8%)	2(1.0%)	1(0.5%)	9(4.8%)
R0	0	1	0	0	1
R0a	0	0	1	0	1
R0a1a	0	0	0	1	1
R0a2'3	0	0	1	0	1
R2	1	0	0	0	1
R7	0	0	0	1	1
R (Total)	1(0.5%)	1(0.5%)	2(1.0%)	2(1.0%)	6(3.0%)
W	0	0	1	0	1
W3b	0	0	1	0	1
W + 194	0	0	0	1	1
W (Total)	0(0.0%)	0(0.0%)	2(1.0%)	1(0.5%)	3(1.5%)
X2e2a	0	0	0	3	3
X2e2c1	0	1	0	0	1
X (Total)	0(0.0%)	1(0.5%)	0(0.0%)	3(1.5%)	4(2.0%)
I1a	1	0	0	0	1
I5	0	0	0	1	1
I (Total)	1(0.5%)	0(0.0%)	0(0.0%)	1(0.5%)	2(1.0%)
D4g1b	1	0	0	0	1
D5a2a	0	0	1	0	1
D (Total)	1(0.5%)	0(0.0%)	1(0.5%)	0(0.0%)	2(1.0%)
C4a1a	0	0	0	1	1
C (Total)	0(0.0%)	0(0.0%)	0(0.0%)	1(0.5%)	1(0.5%)
L2a1 + (16192) *2	0	0	1	0	1
L (Total)	0(0.0%)	0(0.0%)	1(0.5%)	0(0.0%)	1(0.5%)

###  PCA Analysis

**Figure 2:**
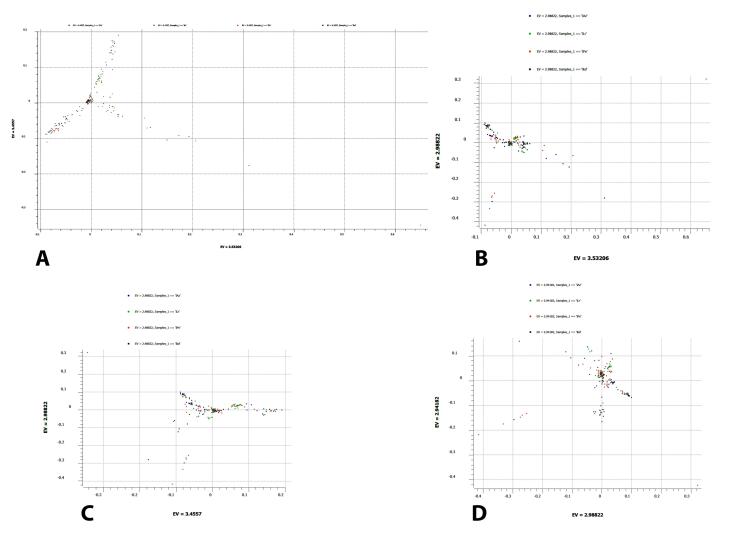
Principal Component Analysis of the Mitochondrial DNA from 192 Iranian Samples. The four colors show ethnic groups (IAz: Azeris, ILr: lurs, Ipe: Persians, IKd: Kurds), representing each sample’s variant origin. (A) The X- and Y-axes display principal component 1 (PC1) and principal component 2 (PC2), along with their respective variations percentage or eigenvalues (EV). (B) PC1-PC3, (C) PC2-PC3, and (D) PC3-PC4 are shown as separate scatter plots

 To evaluate the variation observed in mtDNA attributable to ethnic groups, PCA was conducted on 192 samples (Figure 2). Each principal component accounts for a specific percentage of the total variance in the dataset. The PC1 on the X-axis explains 3.5% of the variance, while PC2 on the Y-axis accounts for 3.4%, resulting in a cumulative variance of 6.9% (Figure 2A). Additionally, PC1 vs. PC3 has a low percentage of 3.5% on the X-axis, 2.9% on the Y-axis (Figure 2B), PC2 vs. PC3 has 3.4% on the X-axis and 2.9% on the Y-axis (Figure 2C), and PC3 vs. PC4 has 2.9% on the both X-axis and Y-axis (Figure 2D). The components in all four scatter plots demonstrate low variance within the dataset ( < 80%‒90%), indicating minimal differences among the original variables across ethnic groups.

## Discussion

 This study aims to elucidate the genetic structure of the Iranian population on the complete mitochondrial genomes of 192 individuals. The Iranian plateau features a wide range of genetic diversity with 15 primary haplogroups. Most haplogroups (H, U, HV, J, K, and T) are typical of West Eurasia. The most prevalent haplogroups, U (22.4%) and H (20.3%), suggest a genetic connection with southwest Asia and Europe, where they are found at substantial frequencies. The lack of diversification in haplogroups H and U may be due to a smaller sample size. However, the distribution frequency of these lineages differs among various populations. Three out of four groups show high frequencies of haplogroup H (5.7%, 5.7%, and 6.3%, respectively), but the frequency of haplogroup H is the lowest in Kurd ethnicity (2.6%). The U and J haplogroups are more pronounced in Kurd and Lur groups (8.8% and 6.3%, respectively) than in others. Another notable difference between Kurds and the other three groups studied is the higher prevalence of haplogroup X2 (1.5% vs. 0.5% in Lurs and 0.0% in Persians and Azeris) and T (4.2% vs. 0.5%‒2.6% in the others). In contrast, haplogroup K dominates in Azeris (3.2%), being found only in 0.5%‒1.6% in others. Haplogroups M and D that occur predominantly in today’s populations in Central/East Asia were found only in a few individuals from the Azeri and Persian groups, suggesting low levels of gene flow from Asia into Anatolia and influence specifically on these groups. This aligns with the findings of Quintana-Murci *et al*., who reported the low frequencies of Asian haplogroups in the populations from the Anatolian/Caucasus area and the Iranian Plateau.^35^ A similar observation applies to African-specific lineages, represented by haplogroup L2a which was found in Persians at a frequency of 0.5%. This result is consistent with the findings of Terreros et al and Quintana-Murci et al,^35,36^ who observed similar proportions of the L haplotype (2.6%) in both the southern and northern regions of Iran. This haplotype is now exclusively found in African populations. A unique Latino/mixed American haplogroup C in the Kurdish group indicates diverse genetic influences. We suggest that the current presence of the L and C haplogroups among Iranian people is primarily attributable to geographic factors, including historical migrations. Furthermore, these haplogroups likely originate from ancestral populations that have significantly influenced the present-day populations in this region and beyond. All four Iranian populations represent similar frequencies of the western Eurasian component, shown by haplogroups N, R, and U. However, notable differences exist among some sub-haplogroups, with J1b1b (4.6%) and U7a (6.2%) being the most prevalent in the Iranian population. H haplogroup samples are distributed across 22 sub-haplogroups, showing no similarity between populations. This varied distribution of sub-haplogroups suggests gene flow into Iran from different periods and origins (Table 1).

 In this study, PCA was employed to reduce the dimensionality of the dataset and identify the underlying structure. In the scatter plots (Figures 2A, B, C, and D), the first principal component (PC1) explains the largest portion of variance. The second principal component (PC2) explains the next highest amount of variance, followed by the third and fourth principal components (PC3 and PC4). This indicates that these components capture a small but significant portion of the variability in the data. PC1 and PC2 principal components explained 3.5% and 3.4% of the total variance, respectively, with an accumulative variance of 6.9%. The little variation in the first few PCs indicates little correlation among the original variables. The PCA results reveal distinct patterns in the data similar to the central Iranian cluster (CIC) of the Iranian population. This study aligns with the findings of Mehrjoo et al,^27^ who analyzed autosomal diversity among eleven different Iranian ethnic groups. Their research indicates the existence of a CIC cluster that includes seven ethnicities, including the four ethnicities analyzed in our study (Azeri, Persian, Kurd, and Lur). However, it is important to note that the low percentage of variance explained by the first two PCs suggests that other factors may also influence the dataset. Since mtDNA is inherited only from the mother, it represents a single lineage, which may not capture the full genetic diversity of a population. Also, mtDNA has a relatively small genome and fewer variants than nuclear DNA. This limited variation in mtDNA might result in less information than nuclear DNA analyses. Future studies should consider comparing these ethnicities with ancestral samples to identify the mtDNA structure and origins of the Iranian ethnic groups.

 This study compares two methods for mitochondrial genome analysis: WES and targeted whole mitochondrial sequencing. Previous studies have used WES data for reconstructing human mitogenomes.^21,23,26^ Our research suggests that targeted mitochondrial sequencing, with its superior depth of coverage compared to WES, is an effective method for accurately reconstructing the mtDNA sequence. Based on our comparisons between targeted whole mtDNA sequencing and WES datasets, the average coverage depth of mtDNA for targeted sequencing was approximately 10 times higher than that achieved through WES. Considering the number of variants compared to those identified through targeted sequencing (57.8% WES *vs.* 100% in whole mtDNA sequencing as a gold standard method), several samples showed a loss of variants in WES. This may be due to the low number of mapped mtDNA reads recovered in these samples, leading to a reduced mtDNA coverage depth. However, according to the 2021 study by García-Olivares et al,^37^ the WES method is an alternative cost-benefit method to mtDNA haplogroup evaluation with accurate tools despite a reduced sequencing depth than the targeted sequencing method. Several newly identified variants from the targeted method are not found in the existing mitochondrial population databases. While six of these novel variants could potentially be pathogenic according to the HmtDB database, they are categorized as variants of uncertain significance (VUS) based on the ACMG guidelines. It is crucial to highlight that these results are based exclusively on *in-silico* analyses, and none of the variants have been validated as pathogenic mutations in clinical contexts.

## Conclusion

 Despite the significant diversity among Iranian ethnicities, these findings indicate lower diversity within four ethnic groups of the CIC, a conclusion made possible by the high-throughput sequencing approach used in this study. To determine the maternal origin and thoroughly investigate the various ethnic groups within the Iranian population, we recommended more comprehensive studies to be conducted across other Iranian ethnic groups. Additionally, their genetic variants should be compared with those obtained from ancient Iranian DNA. Also, comparing the mtDNA of Iran and neighboring regions will help trace the patterns of Iranian population migration.
